# Distribution of serum adiponectin isoforms in pediatric patients with steroid-sensitive nephrotic syndrome

**DOI:** 10.1007/s10157-021-02085-w

**Published:** 2021-06-01

**Authors:** Tetsuro Tamai, Kaori Kamijo, Yoshifusa Abe, Satoshi Hibino, Shunsuke Sakurai, Shuichiro Watanabe, Yoshitaka Watanabe, Satomi Nimura, Atsutoshi Shiratori, Takaaki Takayanagi, Tsuneki Watanabe, Yuya Nakano, Hirokazu Ikeda, Kazushige Dobashi, Yasuko Nakano, Katsumi Mizuno, Kazuo Itabashi

**Affiliations:** 1 Department of Pediatrics, Showa University School of Medicine, Tokyo, Japan; 2 Children’s Medical Center, Showa University Koto Toyosu Hospital, 5-1-38 Toyosu, Koto-ku, Tokyo, 135-8577 Japan; 3 Department of Pediatric Nephrology, Aichi Children’s Health and Medical Center, Aichi, Japan; 4 Watanabe Children’s Clinic, Tokyo, Japan; 5 Department of Hospital Pharmaceutics, School of Pharmacy, Showa University, Tokyo, Japan; 6 Department of Pediatric Nephrology, Tokyo Women’s Medical University School of Medicine, Tokyo, Japan; 7 Department of Pediatrics, Tokyo Metropolitan Health and Medical Treatment Corporation Ebara Hospital, Tokyo, Japan; 8 Children’s Medical Center, Showa University Northern Yokohama Hospital, Kanagawa, Japan; 9 Department of Pediatrics, Showa University Fujigaoka Hospital, Kanagawa, Japan; 10 Department of Pediatrics, Enzan Citizen Hospital, Yamanashi, Japan; 11 Drug Treatment Laboratory, Clinical Department, Yokohama University of Pharmacy, Kanagawa, Japan

**Keywords:** Adiponectin, Adiponectin isoform, High-molecular-weight, Nephrotic syndrome, Children

## Abstract

**Background:**

Serum adiponectin circulates in three multimeric isoforms: high-molecular-weight (HMW), middle-molecular-weight (MMW), and low-molecular-weight (LMW) isoforms. Potential change in the circulating adiponectin levels in patients with nephrotic syndrome (NS) remain unknown. This study aimed to assess the levels of total adiponectin and the distribution of its isoforms in pediatric patients with NS.

**Methods:**

We sequentially measured total adiponectin and each adiponectin isoform levels at the onset of NS, initial remission, and during the remission period of the disease in 31 NS patients. We also calculated the ratios of HMW (%HMW), MMW (%MMW), and LMW (%LMW) to total adiponectin incuding 51 control subjects.

**Results:**

The median of total serum adiponectin levels in patients were 36.7, 36.7, and 20.2 μg/mL at the onset, at initial remission, and during the remission period of NS, respectively. These values were significantly higher than those in control subjects. The median values of %HMW, %MMW, and %LMW values were 56.9/27.0/14.1 at the onset, 62.0/21.8/13.4 at the initial remission, and 58.1/21.7/17.5 at during the remission period of NS, respectively. Compared with control subjects, %HMW at initial remission and %MMW at the onset were high, and the %LMW values at the onset and at initial remission were low.

**Conclusions:**

In patients with NS, total serum adiponectin levels increase at the onset of the disease, and the ratio of adiponectin isoforms changes during the course of the disease. Further studies are needed to delineate the mechanisms between proteinuria and adiponectin isoforms change.

## Introduction

Adiponectin is an adipocyte-secreted protein that positively affects insulin sensitivity and coronary artery disease [1, 2]. Hypoadiponectinemia is associated with conditions such as insulin resistance, obesity, metabolic syndrome, type 2 diabetes mellitus, hypertension, dyslipidemia, and atherosclerosis [2, 3]. Besides the reported associations in adults, plasma adiponectin levels in children are associated with several diseases, including obesity, insulin resistance, and Kawasaki disease [4–6].

In kidney diseases, the roles of adiponectin remain poorly understood; furthermore, results from different studies are controversial. Low adiponectin is correlated with albuminuria in both mice and humans [7]. Adiponectin knockout in mouse models leads to increased podocyte injury and albuminuria, whereas adiponectin therapies restore the podocyte foot processes [8]. However, in humans, significantly elevated serum adiponectin levels are reported in patients with chronic kidney diseases (CKD) or end-stage renal disease (ESRD) as well as in those who received dialysis or kidney transplants [9, 10]. In addition, serum and urine adiponectin levels were positively correlated in both children and adults with focal segmental glomerulosclerosis [11]. Interestingly, patients with nephrotic syndrome (NS) with normal renal function have markedly increased serum adiponectin levels [12, 13]. Furthermore, it was recently reported that adiponectin is one of the candidate biomarkers with potential to identify mechanistic molecular pathway and targets of steroid resistance [14]. However, little is known about the mechanism of adiponectin upregulation in NS.

Adiponectin circulates in the form of three oligomeric complexes: high-molecular-weight (HMW, 400–600 kD, oligomer), middle-molecular-weight (MMW, 180 kD, hexamer), and low-molecular-weight (LMW, 75–90 kD, trimer) isoforms [1, 15]. In human serum, the percentages of HMW, MMW, and LMW in total adiponectin are approximately 40–50%, 25–35%, and 25%, respectively [1, 16]. It has been suggested that the proportion of each sub-fraction determines the biological activities of adiponectin [17].The proportions of HMW and MMW are higher in females than in males [18]. Serum HMW adiponectin levels may be a better marker for predicting insulin resistance, metabolic syndromes, endothelial dysfunction, obese children, and type 2 diabetes mellitus than total adiponectin levels [17, 19]. The HMW-to total adiponectin ratio may be positively associated with aortic stiffness in patients undergoing hemodialysis [20]. However, the effects of NS on the proportions of the adiponectin sub-fractions remain unknown. Therefore, we aimed to evaluate the effects of the nephrotic state on serum adiponectin levels and levels of each adiponectin isoform in patients with steroid-sensitive NS.

## Materials and methods

Thirty-one pediatric patients (23 males and 8 females) with newly diagnosed steroid-sensitive NS were admitted to our hospital from March 2006 to July 2019. Patients with steroid-resistant NS were excluded. Drawing blood is somewhat invasive in children. To solve difficulty in obtaining blood samples from entirely healthy children, overall, 51 subjects without clinically detectable kidney dysfunction (28 males and 23 females) were also enrolled in this study as control ones at authors’ outpatient clinic. To measure serum adiponectin level in all of them, blood was drawn from January 2011 to September 2019. Written informed consent was obtained from either one or both parents of each child before the enrollment in the study. This study was approved by the ethics committee of the Showa University School of Medicine (No. 759, No. 20T5017, No. 3387), and the study was performed according to the ethical standards of the 1964 Helsinki Declaration and its later amendments.

The following conditions presented in the control patients: seven were examined for health check-up; hydronephrosis and immunoglobulin A (IgA) vasculitis (including one patient with nephritis) in five patients each; asymptomatic hematuria and mycoplasmal infection in four patients each; acute pneumonia in three patients; acute otitis media, IgA nephropathy, simple goiter, and urticaria in two patients each; abnormal urinalysis, cervical lymphadenopathy, Hirschsprung disease, immune thrombocytopenic purpura, iron deficiency anemia, Kawasaki disease, neutropenia, nocturnal enuresis, protein losing enteropathy, renal scar, subcutaneous abscess, sepsis, thin basement membrane disease, thrombocytopenia, and vesicoureteral reflux in one patient each. Two control subjects had mild proteinuria with a urinary protein-to-creatinine ratio (P/Cr) < 0.5 g/gCr, and six control subjects with Hirschsprung disease, IgA vasculitis, iron deficiency anemia, Kawasaki disease, protein losing enteropathy, and sepsis had hypoalbuminemia (< 2.5 g/dL) without proteinuria.

The definition and criteria of NS used here are based on the clinical practice guidelines for pediatric idiopathic nephrotic syndrome 2013 [21]. A patient was considered to have NS if the proteinuria was ≥ 40 mg/h/m^2^ at night urine collection and the serum albumin level was ≤ 2.5 g/dL. Complete remission was defined as the qualitative resolution of proteinuria (negative by dipstick method) in the early morning urine for 3 consecutive days. Relapse was defined as positive results of protein (≥ 2 +) in the early morning urine for 3 days after remission. Steroid-sensitive NS was defined as NS conditions that completely remitted within 4 weeks after initiating of daily prednisolone therapy. According to the clinical practice guidelines for pediatric idiopathic nephrotic syndrome 2013, prednisolone treatment consisted of 60 mg/m^2^/day or 2 mg/kg/day in three divided doses daily for 4 weeks, followed by 40 mg/m^2^/day or 1.3 mg/kg once in the morning on alternative days for 4 weeks, or 40 mg/m^2^ or 1.3 mg/kg once in the morning on alternate days, continued for 2–6 months with tapering of the dose. [21].

Serum samples were stored at − 80 °C until the time of adiponectin measurement. The levels of total serum adiponectin and the three adiponectin isoforms were analyzed using the enzyme-linked immunosorbent assay method and the Human Adiponectin ELISA Kit for total and multimers (Sekisui Medical Co. Ltd., Tokyo, Japan). To investigate the changes in the distribution of adiponectin isoforms at the onset of NS (before initiation of prednisolone treatment), at initial remission (just after detecting negative proteinuria by dipstick method), and the remission period after the initial remission of the disease, the ratios of HMW (%HMW), MMW (%MMW), and LMW (%LMW) to total adiponectin were calculated for each time point.

Statistical analyses were performed using Prism 8 (GraphPad, San Diego, CA). Data are expressed as median (interquartile range). Analyses included chi-square test, Mann–Whitney *U* test, Kruskal–Wallis test, and Spearman’s rank correlation coefficient. *P* values < 0.05 were considered significant.

## Results

The clinical and biochemical characteristics for control subjects and for patients with NS are summarized in Table 1. At the onset of NS, the median age of the 31 patients was 3 years (1–6). The median duration of proteinuria after admission until the first day of initial remission was 9 days (7–11). In two patients, serum creatinine levels were slightly elevated at the onset of NS. The median interval between the onset of NS and the third stage (the remission period after the initial remission) was 23 days (13–42). No significant differences were found in ages or male-to-female ratios of the two groups (Mann–Whitney *U* test or chi-square test). Total protein and serum albumin levels in patients with NS were significantly lower at the onset and the initial remission stages than those in the control subjects. In contrast, total cholesterol levels were significantly higher at all three stages of the disease, and the triglyceride levels were significantly higher in patients at onset and in initial remission stages than in control subjects. No significant difference was found in the leptin levels between the two groups. There was significantly negative correlation between interval from onset and total serum adiponectin level (*P* < 0.01), while there were no significant correlations between interval from onset and multimeric isoform ratios by Spearman’s rank correlation coefficient test. After the treatment of initial NS, renal biopsies were performed in five patients because of frequent relapses of NS during their clinical courses. Minor glomerular abnormalities were found in all five patients during microscopic examinations.

**Table 1:** Clinical characteristics and biochemical data of control subjects and patients with nephrotic syndrome

Control subjects			Nephrotic syndrome patients					
Age (years)	5 (2–11)	(*n* = 51)	3 (1–6)	(*n* = 31)				
Male: female	28:23		23:8					
			Onset		Initial remission		Remission period	
Blood urea nitrogen (mg/dL)	12.0 (8.7–15.7)	(*n* = 46)	11.7 (9.5–16.0)	(*n* = 31)	9.4 (9.5–16.0)	(*n* = 31)	12.4 (11.6–15.4)	(*n* = 30)
Creatinine (mg/dL)	0.3 (0.22–0.46)	(*n* = 46)	0.27 (0.20–0.30)	(n = 31)	0.24 (0.20–0.30)	(*n* = 31)	0.27 (0.20–0.31)	(*n* = 30)
Total protein (mg/dL)	7.1 (6.5–7.6)	(*n* = 45)	4.0 (3.6–4.4)	(*n* = 31)****	5.1 (3.6–4.4)	(*n* = 31)****	6.4 (6.0–6.6)	(*n* = 31)
Albumin (mg/dL)	4.4 (4.0–4.5)	(*n* = 36)	1.1 (1.0–1.4)	(*n* = 31)****	2.1 (1.0–1.4)	(*n* = 31)****	3.3 (3.0–4.0)	(*n* = 31)
Total cholesterol (mg/dL)	160 (135–188)	(*n* = 35)	424 (344–485)	(*n* = 31)****	453 (344–485)	(*n* = 26)****	266 (210–326)	(*n* = 30) ***
Triglyceride (mg/dL)	83 (62–127)	(*n* = 35)	191 (162–312)	(*n* = 31)****	236 (162–312)	(*n* = 22)****	135 (72–211)	(*n* = 27)
Leptin (mg/dL)	5.4 (3.3–8.1)	(*n* = 46)	3.9 (3.2–5.9)	(*n* = 30)	5.2 (3.2–5.9)	(*n* = 30)	6.1 (4.0–9.2)	(*n* = 30)

^***^P < 0.001 and ****P < 0.0001 using Kruskal–Wallis test, followed by Dunn’s multiple comparison test

**Fig. 1:**
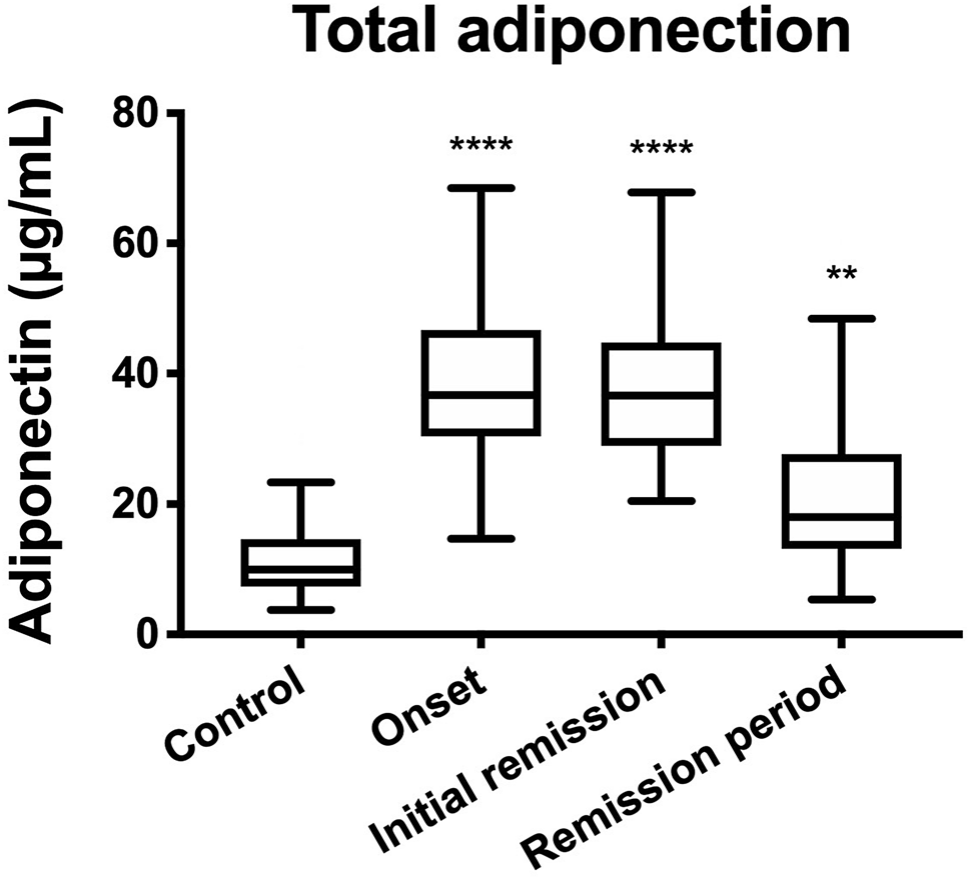
Median total adiponectin levels. Statistical significance was calculated using Kruskal–Wallis test, followed by Dunn’s multiple comparisons test. **P < 0.01, and ****P < 0.0001

**Fig. 2:**
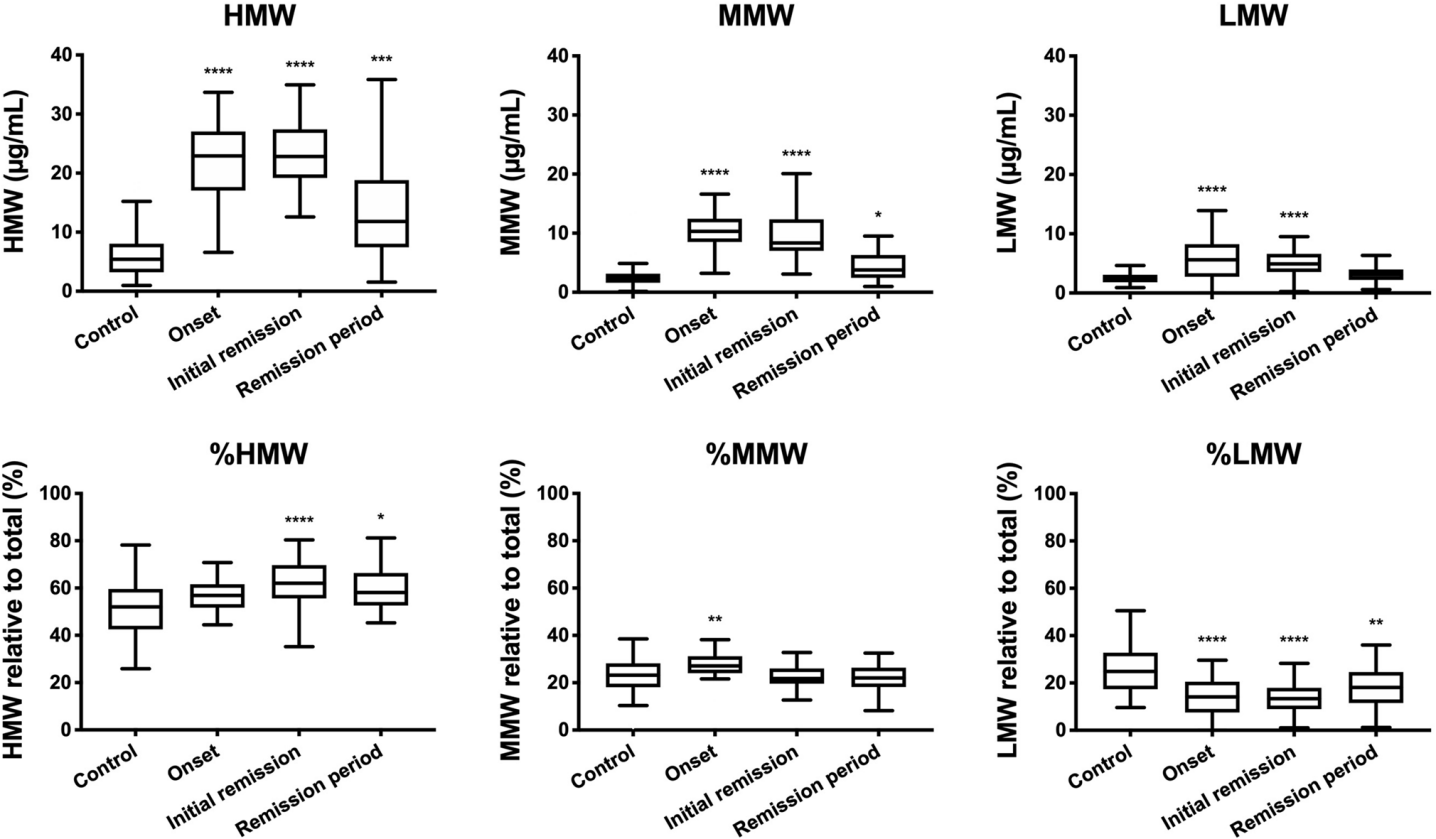
Median adiponectin isoform levels. *HMW* high-molecular-weight adiponectin; *MMW* middle-molecular-weight adiponectin; *LMW* low-molecular-weight adiponectin; *%HMW* ratio of HMW to total adiponectin; *%MMW* ratio of MMW to total adiponectin; *%LMW* ratio of LMW of total adiponectin. Statistical significance was calculated using Kruskal–Wallis test, followed by Dunn’s multiple comparisons test. *P < 0.05, **P < 0.01, ***P < 0.001, and ****P < 0.0001

Total serum adiponectin levels were 36.7 (30.4–46.7) μg/mL, 36.7 (28.9–44.8) μg/mL, and 20.2 (13.2–32.1) μg/mL at the onset of NS, at initial remission, and during the remission period after initial remission of NS, respectively. In 51 control subjects, the total serum adiponectin level was 10.0 (7.4–14.6) μg/mL (Fig. 1). HMW adiponectin levels in the above 3 stages were 22.9 (17.0–27.0) μg/mL, 22.8 (19.2–27.4) μg/mL, and 12.0 (7.45–18.8) μg/mL, respectively. In addition, MMW levels were 10.3 (8.5–12.4) μg/mL, 8.3 (7.0–12.4) μg/mL, and 3.8 (2.5–6.9) μg/mL, respectively. Finally, LMW levels were 5.6 (2.8–8.2) μg/mL, 4.9 (3.6–6.6) μg/mL, and 3.1 (2.2–5.3) μg/mL, respectively. Regarding the proportions of adiponectin isoforms at the above three stages, %HMW were 56.9 (51.7–61.6) %, 62.0 (55.7–69.7) %, and 58.1 (52.7–66.4) %; %MMW were 27.1 (24.0–31.2) %, 21.8 (19.6–26.0) %, and 21.7 (16.3–26.4) %; and %LMW were 14.1 (7.5–20.5) %, 13.4 (9.0–18.0) %, and 17.5 (11.2–24.6) %. In those 51 control subjects, HMW, MMW, and LMW levels were 5.4 (3.21–8.0) μg/mL, 2.3 (1.6–3.1) μg/mL, and 2.5 (1.8–3.1) μg/mL, respectively. The %HMW, %MMW, and %LMW were 52.0 (42.5–59.6) %, 23.2 (18.1–28.2) %, and 24.9 (17.4–32.8) %, respectively (Fig. 2).

Total adiponectin levels were significantly higher in patients in all three stages of the disease than those in control subjects. Even excluding control subjects with kidney diseases such as abnormal urinalysis (one patient), asymptomatic hematuria (four patients), IgA vasculitis with nephritis (one patient), IgA nephropathy (two patients), and thin basement membrane disease (one patient), the total adiponectin levels were significantly higher at all three stages of the disease (data not shown).

To investigate whether hypoalbuminemia is related to increased adiponectin levels, total serum adiponectin levels in six patients with hypoalbuminemia (< 2.5 g/dL) without proteinuria were compared with those in patients with NS. In those six patients, total serum adiponectin level was 12.7 (3.8–33.0) μg/mL. The total serum adiponectin level was significantly elevated at the onset of NS in patients with NS (*P* < 0.01, Mann–Whitney *U* test).

Regarding adiponectin isoform levels in patients compared with those in control subjects, HMW, MMW, and LMW were all significantly higher (*P* < 0.0001) at onset and initial remission of NS. During the remission period, significantly higher HMW (*P* < 0.001) and MMW (*P* < 0.05) levels were observed. Regarding the proportion of the adiponectin isoforms, as compared with the control, %HMW was significantly higher at the initial remission stage (*P* < 0.0001) and during the remission period (*P* < 0.05), %MMW was significantly higher at the onset of NS (*P* < 0.01), and %LMW was significantly lower at all three stages (*P* < 0.0001, *P* < 0.0001, and *P* < 0.01, respectively) of NS.

## Discussion

The advantage of this study was measuring both total adiponectin levels and the proportions of adiponectin isoforms, which had not been previously investigated in pediatric patients with NS. In this study, total adiponectin level increased significantly in pediatric patients with NS at the onset and initial remission of the disease compared with that in control subjects. We also observed increases in the levels of HMW, MMW, and %MMW as well as a decrease in %LMW at the onset of NS. These findings suggest that the total adiponectin level and the proportions of the adiponectin isoforms change in NS.

The finding of significantly increased adiponectin levels in patients with NS is consistent with that of previous reports [12, 13]. Zoccali et al. reported that the levels of serum adiponectin levels significantly increased in adult patients with NS secondary to different etiologic causes and were strongly related to the degrees of proteinuria [12]. Similarly, Bakkaloglu et al. reported that serum adiponectin levels remarkably increased in steroid-sensitive NS relapse compared with those in steroid-sensitive NS remission in pediatric patients [13]. The increase in adiponectin level parallels the progression of CKD, and high adiponectin levels are found in patients with ESRDs [22, 23]. In patients with CKD, uremia may reduce adiponectin clearance due to renal dysfunction and lead to impaired biodegradation and abolition [9]. However, adiponectin levels increase in patients with NS despite normal renal function. Therefore, uremic conditions and reduced clearance are unlikely to be associated with the increased adiponectin in NS.

The mechanism for elevated serum adiponectin levels and the direct effects of adiponectin on podocytes in the nephrotic state in NS remain unknown. However, increased serum adiponectin levels were unlikely to be associated with renal dysfunction because most patients with NS maintain renal functions. In urine, adiponectin exists largely as a trimer (LMW), which has a similar or lesser molecular weight than that of albumin [24–26]. Increased glomerular permeability due to podocyte dysfunction can cause hyper-filtration of adiponectin into urine. Hence, the changes in the distribution of adiponectin isoforms observed in our study may be associated with LMW leakage from the kidney. Recent evidence in mice suggests that the adiponectin regulates albuminuria and exerts a protective effect on the podocytes [8]. The adiponectin receptor (AdipoR1) is expressed at a high level in the podocytes and adiponectin signaling via adenosine monophosphate-activated protein kinase (AMPK) regulates NADPH oxidase 4 (Nox4) expression, which is linked to oxidative stress, the fusion of podocyte foot processes, and albuminuria [8]. These findings indicate a possibility that the adiponectin is upregulated in NS to attenuate podocyte damages. Zoccali et al. speculated an alternative possibility that the depletion of albumin and protein in NS triggers metabolic changes that may lead to hyperlipidemia and hyperfibrinogenemia in the liver as well as a parallel increase in the synthesis of adiponectin in fat cells [12]. This adaptation would be appropriate to limit the acute inflammatory phase reactions associated with NS [12]. In our study, six control patients had hypoalbuminemia (< 2.5 g/dL) without proteinuria or adiponectin elevation, which indicates that the prominent proteinuria, rather than the hypoalbuminemia was the trigger for the elevating adiponectin levels. Otherwise, elevated adiponectin levels in NS may be caused by an abnormal response to adiponectin in the kidney due to reduced AdipoR1 density, or signal coupling, or the production of an aberrant form of the molecule, as Sethna et al. implied [11]. It is difficult to know whether the increase in adiponectin levels occurs before the emergence of proteinuria, and thus, further investigations including animal studies are required to clarify the mechanisms of adiponectin upregulation and the its effects on the podocyte in NS. Particularly, to clarify whether postulated podocyte damage is associated with adiponectin receptor density or not, some studies on immunoreactivity of it in kidney biopsy specimens obtained from patients with SSNS and the other glomerular diseases should be conducted.

There are few reports available on adiponectin isoform levels. Nishimura et al. reported that there is no sex-related differences in adiponectin isoform levels and total serum adiponectin, HMW, MMW, and LMW levels are 5.5–6.4 µg/mL, 2.2–3.1 µg/mL, 1.6–1.8 µg/mL, and 1.3–1.7 µg/mL, respectively, in prepubertal school-age children [27]. Compared with those values, some of our patients with NS had higher levels for all adiponectin isoforms. Regarding the association between the adiponectin isoforms and diseases, the HMW levels may serve as a predictor of future cardiovascular events in patients with coronary artery disease, and the HMW-to-total adiponectin ratio may be positively associated with aortic stiffness in patients undergoing hemodialysis [1]. In addition, physiological activities, such as vascular protective activities and the insulin sensitivity, are also affected by HMW or %HMW [20]. Pajvani et al. reported that the HMW-to-total serum adiponectin ratio is useful for monitoring the improvement of insulin sensitivity in response to thiazolidiones in cases of type 2 diabetes [28]. Goto et al. reported that decreases in LMW, HMW, and total adiponectin levels are associated with diabetes [29]. To the best of our knowledge, the changes in the proportion of adiponectin isoforms in NS have not been investigated previously. In our study, both HMW and the %HMW increased in patients with NS, and thus, both HMW and %HMW may promote the protection of podocytes. Recently, it was reported that adiponectin is one of the candidate biomarkers with potential to identify mechanistic molecular pathway and targets or steroid resistance [14]. Therefore, to clarify the mechanism of adiponectin upregulation in NS may lead to exploring its cause and reducing steroid resistance.

This study has some limitations. First, all patients with NS were treated with steroids, and steroid treatment could affect adiponectin levels. Fallo et al. reported that the glucocorticoids decreased plasma adiponectin levels in males, as shown in both healthy subjects receiving acute exogenous administration and patients with chronic endogenous hypercortisolism [30]. By contrast, steroids have an inhibitory effect on TNF-α, which decreases adiponectin level [31]. Uchida et al. reported that adiponectin levels were significantly augmented after glucocorticoid pulse therapy in patients with IgA nephropathy [32]. Furthermore, some reports have shown that steroids have no effect on the adiponectin level [33, 34]. As mentioned above, the effects of steroid treatment on adiponectin are somewhat controversial, but many of studies suggest that steroid treatment increases adiponectin expression [35]. We emphasize that high serum adiponectin levels at the onset were under untreated condition and that all patients with NS were treated with steroids. Second, urine adiponectin levels were not measured in our study. The distribution of adiponectin isoforms in blood may differ from that in urine. In an experimental model, it is reported that the glucocorticoid-mediated tightening can reduce the flux of adiponectin across endothelial monolayers, possibly owing to alterations in the expression profiles of the tight junction proteins [36]. Therefore, the amount of each adiponectin isoform leaked from the kidney may be altered by the steroid treatment. Third, total adiponectin levels were significantly higher in patients in all three stages of the disease than those in control subjects, while there was significantly negative correlation between interval from onset and total serum adiponectin level. The interval may be insufficient to decrease adiponectin level at remission period equal to those of controls. Finally, only a small number of participants were enrolled in this study. Larger-scale studies are needed to further delineate the mechanisms between proteinuria and adiponectin complexes.

In conclusion, elevated serum adiponectin levels in patients with NS are believed to be secondary to proteinuria, but the mechanism remains unknown. This study suggests that total adiponectin levels and proportions of adiponectin isoforms change in the course of NS. A regulatory mechanism may exist between adiponectin and proteinuria.

## Data Availability

Our database is available from the corresponding author on reasonable request.
